# Mechanisms of action of TNF-α and IL-6 in cancer pain

**DOI:** 10.3389/fpain.2026.1981570

**Published:** 2026-10-29

**Authors:** Xin Jin, Jie Liu, Dianqun Ren, Chen Zhang, Zhili Guo, Fang Yu

**Affiliations:** 1 Jiaxing Graduate Joint Training Base, Zhejiang Chinese Medical University, Jiaxing, China; 2 Jiaxing Hospital of Traditional Chinese Medicine, Jiaxing, China

**Keywords:** cancer pain, cross-regulation, cytokine interaction, interleukin 6, mechanisms of action, tumor necrosis factor-alpha

## Abstract

Cancer pain is a prevalent and often treatment-resistant complication of advanced cancer, highlighting the need for mechanism-based non-opioid targets. Inflammation within the tumor microenvironment is central to cancer pain, with TNF-α and IL-6 consistently implicated across preclinical models. However, these cytokines are typically studied as independent mediators. We propose that TNF-α and IL-6 may function as an integrated, putatively self-amplifying inflammator*y* axis. Acting through distinct receptor systems, TNFR1/TNFR2 and IL-6R/gp130, they converge on NF-*κ*B, MAPK, and PI3K-Akt signaling to drive transcriptional amplification, glial activation, and neuronal sensitization. Proposed reciprocal positive feedback and shared regulation of nociceptive effectors, including COX-2, TRPV1, TRPA1, and Nav1.8, may couple this axis to bone destruction, nerve injury, and tumor compression, thereby converting local inflammation into persistent pain. However, direct evidence for a self-amplifying circuit and synergistic analgesia in cancer pain remains limited. The integrated circuit and its proposed effects on cancer pain are inferred relationships that require experimental validation. Most mechanistic evidence derives from bone cancer pain and chemotherapy-induced neuropathy models, whereas several pathway-level observations rely on non-cancer models; accordingly, direct support across the broader spectrum of cancer pain remains limited. Although preclinical blockade of either cytokine attenuates cancer pain, direct causal evidence for synergy remains sparse, and systemic immunosuppression poses a major translational barrier. We argue that comparative single- versus dual-target blockade, combined with sex- and phenotype-stratified designs and local or time-limited delivery strategies, will be essential to determine whether the TNF-α/IL-6 axis is a precision non-opioid target in refractory cancer pain.

## 1 Introduction

Cancer pain is a common and debilitating complication of malignancy. A meta-analysis of 277 studies reported an overall prevalence of 44.5%, with 40.7% of patients with advanced or metastatic cancer experiencing moderate-to-severe pain (1). As cancer survival improves and tumor burden increases, the clinical burden of cancer pain is expected to grow. Opioids remain a mainstay of cancer-pain management and provide effective analgesia for many patients. However, a subset of patients obtains inadequate relief or experiences dose-limiting adverse effects, including tolerance, addiction risk, respiratory depression, and constipation; this is particularly relevant for neuropathic or bone metastasis-related pain (2).

Inflammatory processes within the tumor microenvironment are increasingly recognized as key drivers of cancer pain initiation, maintenance, and chronification. Tumor cells, infiltrating immune cells, and glial cells release pro-inflammatory cytokines that sensitize peripheral and central nociceptive pathways (3). Among these cytokines, tumor necrosis factor-α (TNF-α) and interleukin-6 (IL-6) have attracted particular attention because their pro-nociceptive activities span peripheral sensitization, central sensitization, and neuropathic remodeling, and their levels correlate with pain indices across multiple cancer pain models (4–7).

Here, we synthesize evidence that TNF-α and IL-6 function as an integrated, putatively self-amplifying inflammator*y* axis rather than as parallel mediators. This framing carries testable implications: reciprocal transcriptional induction predicts that blockade of a single cytokine may be partly compensated by the other, meaning that single-agent effects do not necessarily reflect the contribution of the pair, and that studies manipulating one cytokine while measuring the other as a downstream readout cannot be treated as independent lines of evidence. Whether this coupling confers any advantage to combined blockade in cancer pain remains an open question that we address in Section 7. We review their receptor-specific signaling, convergent downstream cascades, positive feedback loops, and pathological coupling to bone destruction, nerve injury, and tumor compression. We then evaluate the therapeutic potential of targeting this axis and highlight key translational challenges, including immunological safety, the absence of causal evidence for synergy, and the need for comparative dual-target and sex-stratified studies to enable precision analgesia.

## 2 Methods

This narrative review drew on PubMed and Web of Science searches (inception to 28 August 2026) using combinations of cancer pain terms (“cancer pain”, “bone cancer pain”, “cancer-induced bone pain”, “chemotherapy-induced peripheral neuropathy”) with TNF-α (“TNF-α”, “TNFR1”, “TNFR2”) and IL-6 (“IL-6”, “IL-6 receptor”, “gp130”, “JAK”, “STAT3”) terms. The 28 August 2026 cutoff applies to the database searches; a small number of references added during revision from citation tracking and reviewer suggestions may postdate this cutoff. We prioritized studies using cancer or cancer-treatment models with behavioral pain endpoints, or receptor/pathway-specific manipulations in nociceptive tissues (dorsal root ganglion, spinal cord, peripheral nerve). Non-cancer models and tumor studies without pain endpoints were retained only when they clarified mechanism and were labeled accordingly. Conflicting and negative findings were not excluded and are noted where reported. This is a mechanistic synthesis rather than an effect estimation; accordingly, no quantitative pooling was performed; coverage is not exhaustive, and conclusions about effect frequency or magnitude cannot be drawn.

## 3 Pathophysiology of cancer pain: inflammation as a driver and regulator

### 3.1 Definition and classification of pain and the mixed mechanisms of cancer pain

Pain is defined by the International Association for the Study of Pain (IASP) as “an unpleasant sensory and emotional experience associated with, or resembling that associated with, actual or potential tissue damage” (8). Mechanistically, pain is classified into nociceptive pain, arising from activation of peripheral nociceptors by tissue injury; neuropathic pain, caused by lesion or disease of the somatosensory nervous system; and nociplastic pain, reflecting altered nociceptive processing without identifiable tissue or nerve damage (9, 10). In the canonical ascending pathway, noxious stimuli activate peripheral nociceptors, generating action potentials that propagate along primary afferent fibers, whose cell bodies reside in the dorsal root ganglia, to synapse in the dorsal horn; signals are then relayed via ascending tracts, principally the spinothalamic tract, to the thalamus and subsequently to somatosensory and limbic cortices, where sensory-discriminative and affective components are encoded (11). In cancer, these categories are not mutually exclusive: tumor- and immune-derived mediators can produce overlapping nociceptive, neuropathic, and nociplastic features, underscoring the need for an integrated mechanistic framework.

Unlike many non-cancer pain conditions, cancer pain is not a single entity: its clinical presentation varies markedly with tumor type and treatment, reflecting distinct underlying pathobiology (12). Within the IASP framework, chronic cancer-related pain is classified into nociceptive and neuropathic categories (13); the nociplastic descriptor, which requires the absence of identifiable tissue or nerve damage, is difficult to apply while cancer is active. These categories frequently overlap, and mixed pain is the most common clinical phenotype in chronic cancer pain (14, 15). Nociceptive cancer pain arises from persistent activation of nociceptors by tumor invasion, local ischemia, bone destruction, and treatment-related tissue injury from surgery, radiotherapy, or chemotherapy (16). Neuropathic cancer pain, by contrast, results from direct injury to the nervous system, including tumor infiltration of nerves, chemotherapy-induced axonal degeneration and microtubule destabilization, and radiation-induced nerve fibrosis, leading to ectopic discharge and sensitization of nociceptive pathways (17). Central sensitization, which also occurs within nociceptive and neuropathic cancer pain, should not be equated with the nociplastic descriptor (18).

### 3.2 Pro-nociceptive role of inflammation

Despite etiological heterogeneity, cancer pain phenotypes converge on inflammatory signaling as a shared pathological driver. Peripherally, tumor microenvironment-derived cytokines act directly on nociceptor terminals, reducing activation thresholds so that ordinarily innocuous mechanical and chemical stimuli elicit pain (19). Centrally, sustained inflammatory input activates spinal glia, producing central sensitization and maladaptive neural remodeling (5, 6). This inflammatory state is also self-perpetuating: tumor cells release IL-6 and TNF-α through aerobic glycolysis, oncogenic signaling, and necrosis (20, 21); infiltrating macrophages and stromal cells further propagate cytokine production (22); these activated immune cells also serve as the cellular interface between tumor and nerve, releasing BDNF, PGE₂, and NGF that act on adjacent nociceptors while sustaining TNF-α/IL-6 production through reciprocal activation (23, 24); and cancer-directed therapies—including surgery (25), chemotherapy (26), radiotherapy (27, 28), and CAR-T cell therapy (29)—impose additional inflammatory injury. The convergence of direct nociceptor sensitization and feed-forward inflammatory remodeling thus positions inflammation as a central mechanism linking heterogeneous cancer pain phenotypes to chronic, treatment-resistant pain.

### 3.3 Inflammation-driven sex and phenotypic differences

Cancer pain-associated inflammation is orchestrated by cytokine networks whose composition and functional impact vary by sex and pain phenotype. Sex is a critical modifier of inflammatory pain mechanisms (30). In non-cancer neuropathic and inflammatory models, TLR4-mediated signaling drives pain primarily in males in a testosterone-dependent manner (31), whereas females rely to a greater extent on spinal cord-infiltrating T cells (32). A parallel divergence is emerging in chemotherapy-induced neuropathic pain: female mice depend on an estrogen-related inflammator*y* axis, while males depend on TLR9-mediated macrophage activation (33). In cancer-induced bone pain (CIBP), however, a systematic review and primary evidence from a CIBP model indicate that many studies have used only one sex, and sex-stratified comparisons remain uncommon (34, 35); consequently, sex-specific immune mechanisms remain largely untested, and conclusions about sex-dependent cytokine biology are incomplete.

Inflammatory mediators are also linked to distinct pain phenotypes. In bone metastatic pain, pro-inflammatory mediators released by hyperactivated osteoclasts sustain persistent background pain and provoke movement-related breakthrough pain by exacerbating bone resorption and microstructural destruction (36, 37). In chemotherapy-induced neuropathic pain, persistent inflammatory signaling in the dorsal root ganglion and spinal cord maintains mechanical allodynia and cold hyperalgesia (38). Collectively, these findings indicate that inflammatory cytokine networks are broadly engaged in cancer pain, but their contributions are shaped by sex and pain phenotype. Although TNF-α and IL-6 are repeatedly detected across cancer pain models and correlate with pain indices, whether their signaling is organized along these same dimensions remains poorly defined, an unresolved issue that limits the design of mechanism-based interventions.

## 4 Dysregulated expression of TNF-α and IL-6 in cancer pain

TNF-α and IL-6 are pleiotropic pro-inflammatory cytokines that regulate acute-phase protein expression, cell proliferation and differentiation, and host defense (39, 40). In the tumor microenvironment, they are produced mainly by infiltrating immune and stromal cells; within the CNS, activated microglia and astrocytes are the principal sources (41–44). Their expression is consistently elevated across cancer pain contexts and across tumor, systemic, and neural compartments. Most mechanistic evidence derives from bone and pancreatic cancer models, which have validated pain endpoints; whether TNF-α and IL-6 contribute more to pain in these tumors than in others has not been directly compared. TNF-α participates in tumor promotion and progression (40), but whether specific carcinogen exposures initiate TNF-α/IL-6 overexpression has not been examined in the cancer pain literature. In colorectal cancer, tumor tissue secretes higher levels of TNF-α and IL-6 than adjacent non-tumor tissue (45), suggesting a tumor-intrinsic inflammatory program that may contribute indirectly to pain by accelerating tumor progression.

In non-small cell lung cancer, serum TNF-α and IL-6 are higher in patients with severe pain than in those with mild pain or healthy controls (46). In a KPPC pancreatic cancer model, pain-related behavior coincides with elevated plasma TNF-α and IL-6, both of which decline following mirogabalin-induced analgesia (47). In rat cancer-induced bone pain, spinal TNF-α and IL-6 are upregulated relative to sham controls (48).

In multiple myeloma and bone metastases, these cytokines drive osteoclast-mediated bone resorption and persistent pain through RANKL/RANK signaling (49, 50). Cancer treatments similarly induce inflammatory cytokine release: chemotherapy elevates IL-6 in the dorsal root ganglion (51), radiotherapy triggers cytokine release (52), and CAR-T-associated cytokine storm produces rapid IL-6 surges (53), all of which are temporally associated with pain exacerbation. Consistent with a functional role, the receptors TNFR1, TNFR2, IL-6R, and gp130 are expressed in sensory neurons and the tumor microenvironment (54, 55) and correlate positively with pain behavior (56, 57). In a Walker 256 rat model, antagonists targeting IL-1R, IL-6R, or TNFR1 in the midbrain periaqueductal grey attenuated hyperalgesia (58). In the same model, pentoxifylline (an inhibitor of TNF-α synthesis) and SC144 (an inhibitor of gp130-associated signaling) reduced TRPA1 expression and p38/JNK phosphorylation in the dorsal root ganglia (59). These findings support involvement of TNF-related and gp130-associated signaling, but do not establish receptor-specific causality. Together, these data indicate that the TNF-α/IL-6 system is consistently associated with cancer pain and that its receptors are functionally implicated, although whether the two cytokines act as a coupled circuit *in vivo* remains to be directly tested in dedicated dual-blockade studies (Table 1).

**Table 1:** Evidence-context classification of studies on TNF-α/IL-6 in cancer pain.

Reference	Tier	Model/population	Sex	Intervention/target studied	Compartment/ readout	Pain outcome	Tumor/bone outcome	Principal limitation
Liu et al. 2021 46	A	Human NSCLC, *n* = 113; 109 healthy controls	Both sexes; distribution NR	None (observational cytokine assay)	Serum (systemic)	VAS-stratified: IL-1/IL-6/TNF-α higher in severe-pain (VAS >3) than in the mild-pain group (VAS ≤ 3)	All three cytokines independent prognostic risk factors; AUC 0.846 for prediction of poor NSCLC prognosis	Cross-sectional, single center; no causal inference; sex distribution NR
Itaya et al. 2023 47	A	KPPC mouse; PDAC	NR	Mirogabalin 10 mg/kg/day p.o. (α2*δ* ligand; not cytokine-selective)	Systemic plasma cytokines	Hunching and grimace scores improved; plasma TNF-α, IL-6, IFN-*γ* reduced	Tumor growth and Ki-67 increased; survival unchanged	Sex NR; analgesia accompanied by enhanced tumor proliferation; single model
Zhang et al. 2019 58	A	Wistar rat; Walker 256 in tibial marrow	Male	IL-1R, IL-6R and TNFR1 antagonists; gp130 inhibitor SC144; PI3K-mTOR inhibitors	Midbrain Periaqueductal gray (PAG)	Reduced mechanical and thermal hyperalgesia; downregulated p-PI3 K, p-Akt and p-mTOR	Not assessed	Male only; receptor antagonists not subtype-exclusive; no bone endpoint
Zhao et al. 2019 59	A	Wistar rats, bone cancer model	Male	pentoxifylline (TNF-α synthesis inhibition); SC144 (gp130 inhibition)	Dorsal root ganglia (TRPA1, p38/JNK)	Reduced mechanical and thermal hyperalgesia; DRG TRPA1, p38/JNK phosphorylation reduced	Not assessed	Male only; intervention not cytokine-selective
Scheff et al. 2017 67	A	C57BL/6 and immunodeficient mice; OSCC (cell-line supernatant; 4NQO carcinogenesis)	Female	C87 (TNF-TNFR signaling inhibitor)	Tongue; trigeminal afferents	Orofacial functional allodynia abolished; reduced afferent sensitization	T-cell infiltration perturbed	Female only; supernatant model does not support tumor growth
Wang et al. 2020 73	A	Human pancreatic cancer, *n* = 54	Both sexes; distribution NR	None (observational; immunohistochemistry)	Tumor tissue (NF-*κ*B, COX-2)	Pain scores correlated positively with NF-*κ*B and COX-2 expression	Higher pathological stage in patients with more severe pain	No intervention; TNF-α/IL-6 not measured directly; NF-κB/COX-2 used as proxy; sex distribution NR
Constantin et al. 2008 84	A	C57BL/6J mice; hind-limb tumor	NR	Etanercept; TNFR1 and TNFR2 deletion	Hind-limb tumor; nociceptors	Heat hyperalgesia and nociceptor sensitization prevented; TNFR2 effect > TNFR1	Not assessed	Sex not reported; soft tissue rather than bone metastasis model; heat hyperalgesia as the only endpoint
Wang et al. 2011 86	A	Sprague-Dawley rat; Walker 256 in tibial marrow	Female	U0126 (MEK inhibitor); fluorocitrate, intrathecal	Spinal L4-5 (ERK/CREB)	Mechanical hyperalgesia attenuated; p-ERK reduced	Bone destruction confirmed radiologically/histologically (model characterization); treatment effects on tumour/bone not assessed.	Female only; intrathecal; downstream of TNF-α/IL-6
Tang et al. 2022 87	A	Sprague-Dawley rat; Walker 256 in tibial marrow	Female	PAR2 knockdown/inhibitor ± ERK activator and CREB inhibitor, intrathecal	Spinal cord L4-5 (ERK/CREB; astrocyte)	Mechanical and thermal hyperalgesia attenuated; p-ERK and p-CREB reduced	Not quantified (tibial morphology and H&E only)	Downstream of TNF-α/IL-6, not cytokine-selective; female only; intrathecal route
Zhou et al. 2019 91	A	Sprague-Dawley rat; Walker 256 in tibial marrow	Female	XPro1595, intrathecal (selective soluble-TNF inhibitor)	Spinal dorsal horn (glia; p38 MAPK)	Dose-dependent analgesia; ED_50_ 9.69 mg/kg; glial activation and TNF-α/IL-1β/IL-6 reduced	Not assessed	Female only; intrathecal delivery; tumor burden not quantified
Wang et al. 2012 92	A	Wistar rat; Walker 256 in tibial marrow	Female	SP600125, intrathecal (JNK inhibitor)	Spinal cord	Mechanical allodynia attenuated at day 12; cumulative effect with repeated dosing	Not assessed	Female only; JNK is downstream and not TNF-α/IL-6-specific
Fu et al. 2023 95	A	Mice, 4T1 breast cancer bone pain	Female	GSK690693 (pan-Akt inhibitor; suppresses WNK1)	Spinal dorsal horn; serum	Paw withdrawal threshold increased; TNF-α and IL-17 reduced	Tibial erosion assessed by H&E	Female only; pan-Akt lacks cytokine selectivity
Linher-Melville et al. 2019 113	A	Nude mouse; human breast cancer cells in distal femoral epiphysis	Female	DR-1-55 i.p. for 14 days (STAT3 inhibitor)	Femoral tumor; systemic	Delayed onset and reduced severity of spontaneous and evoked nociception	xCT (SLC7A11), IL-6 and IL-1β mRNA reduced; osteolysis unchanged	Females only, immunodeficient xenograft; no bone protection despite analgesia
Remeniuk et al. 2018 118	A	Fischer 344 rat; MAT B III mammary adenocarcinoma sealed in tibia	Female	TB-2-081 s.c. via osmotic minipump (small-molecule IL-6 antagonist)	Bone exudate and plasma	Prevented ongoing bone pain and evoked hypersensitivity	Tumor growth unchanged; bone remodeling reduced; fracture delayed	Female only; single model; IL-6 antagonist not clinically approved
Lan et al. 2010 119	A	Female Sprague-Dawley rat; Walker 256 in tibial marrow	Female	TLR4 siRNA, intrathecal (day 4 or day 9 after inoculation)	Spinal microglia	Tactile allodynia and spontaneous pain reduced; day 4 prevents, day 9 only attenuates established pain	Not assessed	Female only; TLR4 acts upstream of TNF-α/IL-6 (indirect)
Fang et al. 2015 129	A	Sprague-Dawley rat bone cancer (tibial)	Female	JAK and PI3 K inhibitors; IL-6/sIL-6R trans-signaling blockade	DRG neuron (TRPV1)	Reduced DRG hyperexcitability and hyperalgesia	Not assessed	Female only; kinase inhibitors not IL-6-exclusive; no bone outcome
Yang et al. 2017 141	A	C3H/HeJ mouse; NCTC 2472 osteosarcoma in femoral intramedullary space	Male	Etanercept (TNF-α antagonist; not solTNF-selective)	Spinal cord TNF-α mRNA	Reduced spontaneous lifting, mechanical hyperalgesia and thermal hyperalgesia	Not assessed	Male only; tumor burden and bone destruction not quantified
Tobinick 2003 142— CASE REPORTS	A	Human, *n* = 2; bone metastasis pain (NSCLC; breast)	Both sexes, *n* = 2	Etanercept 25 mg, perispinal s.c.	Spinal metastasis site	Rapid, sustained pain relief at target site	Weight gain in one patient; reduced PET tracer uptake at target site in the other	*n* = 2, uncontrolled and unblinded; no validated pain scale
Liu D et al. 2019 57	B	Bortezomib-induced neuropathic pain	NR	TRPA1 and IL-6 signal inhibition	DRG/spinal	Pain alleviated	N/A (no tumor)	Non-tumor; sex NR
Li C et al. 2018 83	B	Bortezomib-induced neuropathy	NR	Blocking TRPA1 and TNF-α signal	DRG/spinal	Neuropathic pain improved	N/A (no tumor)	Non-tumor chemotherapy model; sex NR
Li SF et al. 2018 112	B	Rat oxaliplatin-induced acute neuropathic pain	NR	AG490 (JAK inhibitor)	Spinal cord	Analgesic effect; spinal IL-6 and p-STAT3 elevated	N/A (no tumor)	Non-tumor chemotherapy model; sex NR; JAK not IL-6-exclusive
El-Sawaf et al. 2024 137	B	Rat vincristine-induced peripheral neuropathy	NR	Melatonin; TNF-α/astrocyte/microglia inhibition	Spinal cord	Pain behavior reduced	N/A (no tumor) (preserved vincristine efficacy in lymphoma cells)	Non-tumor chemotherapy model; sex NR
Fischer et al. 2019 68— NON-CANCER	C	Mice, chronic constriction injury (CCI) neuropathic pain	Males and females; no sex effect	TNFR2 gene deletion; TNFR2 agonist EHD2-sc-mTNFR2; regulatory T-cell depletion	Sciatic nerve/CNS	TNFR2 deletion impaired neuronal regeneration and produced chronic non-resolving pain; TNFR2 agonism promoted long-term recovery	N/A (no tumor)	Not cancer pain
Kim et al. 2016 120— NON-CANCER	C	DBA/1 mouse; experimental collagen-induced arthritis	Female	DTA, dual TNF-α/IL-6 fusion protein	Joint; systemic	Not assessed (arthritis severity only; no pain endpoint)	Reduced joint destruction and bone loss; osteoclastogenesis blocked	Autoimmune model; no nociceptive endpoint
Bolaños et al. 2026 100	D	Cancer cells/tumor immunology	Not applicable (cell line)	TNFR1 expression	Tumor	No pain endpoint	Immune escape and immunosuppressive microenvironment	No pain endpoint
Alam et al. 2024 101	D	Pancreatic tumor model	NR	TNFR1 signaling	Tumor	No pain endpoint	Tumor growth	No pain endpoint
Peng et al. 2015 102	D	Ovarian cancer cells	Not applicable (cell line)	TNFR1/PIK3CB-p110β	Tumor	No pain endpoint	Tumorigenicity	No pain endpoint
Shang et al. 2017 140	D	Human lung cancer cells/metastasis	Not applicable (cell line)	IL-6 and TNF-α (EMT induction)	Tumor	No pain endpoint	Lymph-node and distant metastasis	No pain endpoint; association

Evidence-context classification: This classification describes evidence context, not methodological quality.

Tier A, direct cancer-pain evidence: tumor-bearing humans or animals with at least one pain endpoint reported.

Tier B, cancer-treatment-related evidence without tumor burden at assessment.

Tier C, non-cancer models, labeled NON-CANCER; cited only to clarify mechanism and not counted as cancer-pain evidence.

Tier D, tumor biology without a pain endpoint. Tier is assigned first by model status, then by endpoint status.

Intervention/target studied, the agent, receptor or pathway manipulated or measured. Compartment/readout, the anatomical site of delivery or readout, with molecular or cellular readouts in parentheses where reported. Tumor/bone outcome: “Not assessed” = a tumor or bone compartment was present but no endpoint was measured; “Not applicable (no tumor)” = the model carried no tumor. NR, not reported in the primary publication; N/A, not applicable. CASE REPORTS, uncontrolled observations in individual patients (*n* ≤ 3); they meet the tier A model and endpoint criteria but carry the lowest internal validity.

CNS, central nervous system; COX-2, cyclooxygenase-2; DRG, dorsal root ganglion; ED50, median effective dose; EMT, epithelial-mesenchymal transition; i.p., intraperitoneal; JNK, c-Jun N-terminal kinase; MAPK, mitogen-activated protein kinase; NF-κB, nuclear factor kappa B; NR, not reported; NSCLC, non-small-cell lung cancer; OSCC, oral squamous cell carcinoma; PAG, periaqueductal gray; PDAC, pancreatic ductal adenocarcinoma; PET, positron emission tomography; PI3 K, phosphoinositide 3-kinase; p.o., per os; s.c., subcutaneous; sIL-6R, soluble IL-6 receptor; SLC7A11 (xCT), cystine/glutamate transporter; STAT3, signal transducer and activator of transcription 3; TNFR, TNF receptor; TRPA1, transient receptor potential ankyrin 1; TRPV1, transient receptor potential vanilloid 1; VAS, visual analogue scale. The prefix p- denotes the phosphorylated form.

## 5 Signaling mechanisms of TNF-α in cancer pain

TNF-α is a 17-kDa homotrimeric cytokine that signals through two transmembrane receptors, TNFR1 and TNFR2 (60). TNFR1 is broadly expressed on cancer cells, tumor-infiltrating immune cells, dorsal root ganglion neurons, and spinal glial cells (61, 62); soluble TNF-α preferentially engages TNFR1, recruiting the TRADD/TRAF2 complex to initiate downstream signaling (63). TNFR2 is more restricted under basal conditions but is induced in the tumor microenvironment and in dorsal root ganglion and dorsal horn neurons under pathological conditions (64–66). In an oral cancer model, >90% of lingual primary afferent neurons co-express TNFR1 and TNFR2, indicating robust sensory-neuron upregulation of TNFR2 in disease (67). Both receptors activate NF-*κ*B, MAPK, and PI3 K/Akt cascades, which operate in parallel and undergo crosstalk to form the core signaling hub through which TNF-α is thought to contribute to cancer pain. However, TNFR2 can also exert context-dependent protective effects, promoting Treg-dependent recovery from neuropathic pain in non-cancer models (68). Non-selective TNF blockade cannot distinguish this potentially protective TNFR2 signaling from TNFR1-mediated nociceptive signaling. Mechanistic and pharmacological studies in cancer pain have largely centered on TNFR1; the nociceptive contribution of TNFR2 remains underexplored and resolving whether it plays a net pro- or anti-nociceptive role in cancer pain will require receptor-selective tools.

### 5.1 NF-*κ*B: a central hub for pro-inflammatory transcription

Upon TNFR1 activation, TRADD recruits TRAF2 and RIP-1 (69), activating the IKK complex (70). IKK-mediated phosphorylation and degradation of I*κ*B*α* releases p65/p50 dimers for nuclear translocation, where they initiate transcription of pro-inflammatory genes (71, 72) (Figure 1). This pathway is functionally engaged in cancer pain: NF-*κ*B activation in pancreatic tumor tissues correlates positively with pain scores (73); intrafemoral MDA-MB-231 inoculation in mice upregulates spinal NF-*κ*B and elicits progressive pain (74); and the IKK inhibitor BAY 11-7082 dose-dependently reduces spinal TNF-α and attenuates hyperalgesia in Walker 256 rats (75). These findings are consistent with a positive feedback loop in which TNF-α activates NF-*κ*B and NF-*κ*B sustains TNF-α and broader inflammatory mediator production. The same amplification operates in chemotherapy-induced pain, where paclitaxel (76) or oxaliplatin (77) treatment engages the TNF-α/NF-*κ*B axis and elevates IL-6 and other cytokines.

**Figure 1:**
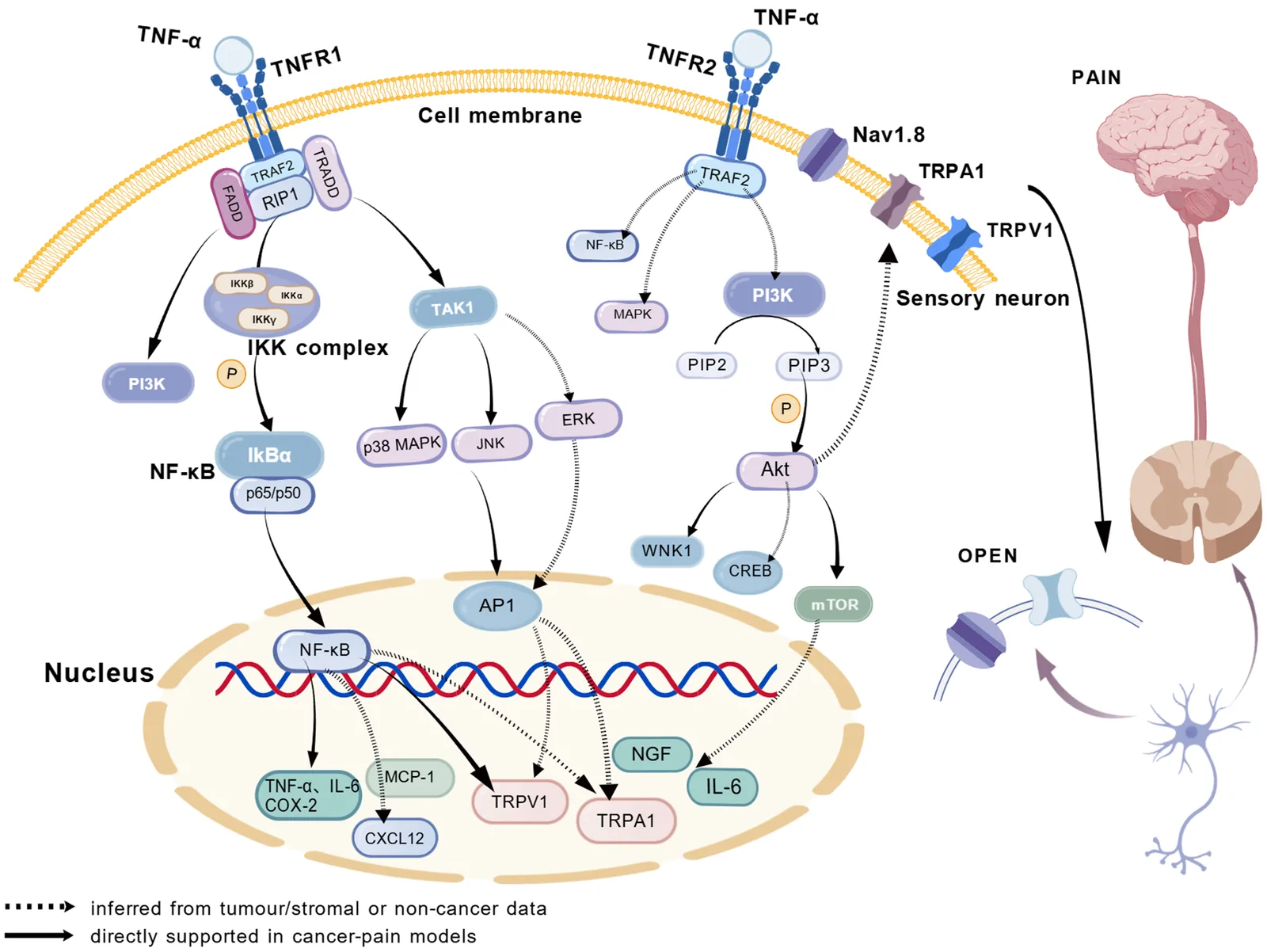
TNF-α signaling network in cancer pain. Figure 1 is a composite schematic that integrates findings from dorsal root ganglion and spinal dorsal horn neurons with glial, tumor, and stromal studies; not every arrow is established specifically in sensory neurons. TNF-α activates TNFR1 and TNFR2. TNFR1 (via TRADD/RIP1/TRAF2) engages IKK–NF-*κ*B and TAK1–MAPK–AP1, upregulating pro-inflammatory mediators and the channels TRPV1 and TRPA1. TNFR2 signals through TRAF2 to NF-*κ*B/MAPK and via PI3K–Akt to mTOR/CREB. TRPV1 is modulated post-transcriptionally by TNFR2-linked p38/PKC, and TRPA1 is induced transcriptionally via NF-*κ*B–HIF1*α*; Nav1.8 is also upregulated. Arrows denoting ERK-driven astrocyte activation are inferred; direct TNF-α → ERK causality in cancer pain has not been established (86, 87). OPEN marks the open (activated) channel state. Solid arrows denote signaling nodes or functional relationships for which direct evidence exists in cancer-pain models. Dashed arrows denote upstream receptor–pathway connections, inferred relationships, or evidence derived from non-cancer, tumour, or stromal studies. A solid arrow downstream does not imply that the entire upstream receptor-to-effector sequence has been demonstrated in cancer pain. Created with BioGDP.com (150). TRADD, TNF receptor-associated death domain; RIP1, receptor-interacting serine/threonine-protein kinase 1; IKK, I*κ*B kinase; TAK1, TGF-*β*-activated kinase 1; AP1, activator protein 1; MAPK, mitogen-activated protein kinase; TRAF2, TNF receptor-associated factor 2; PI3 K, phosphoinositide 3-kinase; Akt, protein kinase B; CREB, cAMP response element-binding protein; mTOR, mechanistic target of rapamycin; NF-*κ*B, nuclear factor *κ*B; HIF1*α*, hypoxia-inducible factor 1*α*; TRPV1, transient receptor potential vanilloid 1; TRPA1, transient receptor potential ankyrin 1; Nav1.8, voltage-gated sodium channel 1.8.

Activated NF-*κ*B converts TNF-α signaling into a coordinated nociceptive program through multiple effectors: it induces CXCL12/CXCR4 to promote mechanical allodynia in bone cancer pain (78, 79); upregulates spinal MCP-1/CCR2, which contributes to breast cancer bone metastasis pain (75, 80); and transcriptionally controls TRPV1, such that naringenin-mediated inhibition of TNF-α/NF-*κ*B signaling reduces TRPV1 expression and alleviates hyperalgesia (81). NF-*κ*B also regulates TRPA1: in rheumatoid arthritis-derived fibroblast-like synoviocytes, TNF-α induces TRPA1 gene expression through NF-*κ*B activation and the downstream transcription factor HIF1*α* (82). In cancer pain models, TRPA1 is upregulated in dorsal root ganglion following bortezomib treatment and in bone cancer pain, and blocking TRPA1 attenuates pain hypersensitivity (59, 83). TNF-α may regulate these two channels through distinct mechanisms: TRPV1 can also be upregulated post-transcriptionally via TNFR2-p38/PKC signaling (84), whereas TRPA1 is induced transcriptionally through NF-*κ*B-HIF1*α* (82). NF-*κ*B therefore functions as a transcriptional hub that amplifies TNF-α input into an integrated expression of cytokines, chemokines, and ion channels, sustaining cancer pain.

### 5.2 MAPK: a core driver of glial activation

The MAPK cascade is a second major TNF-α signaling branch. TNF-α activates MAPK signaling, leading to phosphorylation of ERK, p38, and JNK (85) (Figure 1). Tumor inoculation sequentially activates spinal ERK in neurons, microglia, and astrocytes; MEK inhibition attenuates hyperalgesia and reduces p-ERK (86). PAR2 knockdown also reduces p-ERK/p-CREB and astrocyte activation (87). However, neither study used TNF-α-selective tools, and selective soluble-TNF inhibition with XPro1595 suppressed p38 but not ERK phosphorylation in a bone cancer pain model (91). The proposed TNF-α–ERK link in cancer pain therefore remains inferential. The p38 branch is a well-established mediator of inflammatory and neuropathic pain (88, 89). In contrast to the predominantly central ERK signaling, p38 operates at both peripheral and central sites: TNF-α increases p-p38 immunoreactivity in DRG neurons, and p38 inhibition attenuates mechanical hypersensitivity (90). In the spinal cord, p38 phosphorylation is also increased in glial cells, contributing to dorsal horn glial activation (91). JNK is also engaged: increased phospho-JNK in the spinal cord accompanies mechanical allodynia in bone cancer pain models (92). Together, ERK, p38, and JNK signaling is engaged in cancer-pain models and may contribute to glial activation and central sensitization; however, p38 is supported by direct cancer-pain evidence (91), whereas the TNF-α–ERK link remains inferential.

### 5.3 PI3 K/akt: a direct regulator of neuronal sensitization

PI3 K generates PIP3, leading to Akt activation (93) (Figure 1). TNFR2 signals to PI3 K via TRAF2, whereas TNFR1 activates this pathway indirectly through the TRADD/RIP1 complex (94). In rat models of bone cancer pain, TNFR1 blockade reduces PI3 K activity and attenuates pain behavior (58), indicating that TNFR1 can mobilize PI3 K/Akt despite lacking direct recruitment. In a breast cancer bone metastasis model, spinal p-PI3 K, p-Akt, and TNF-α are concurrently upregulated (95); Akt inhibition with GSK690693 reduces TNF-α and IL-17, suppresses downstream WNK1, and raises mechanical paw withdrawal thresholds, suggesting that PI3 K/Akt not only transduces TNF-α signals but also feeds back to sustain the inflammatory milieu—an additional positive amplification loop.

At the neuronal level, PI3 K/Akt directly couples TNF-α to nociceptor sensitization. Nav1.8 is a tetrodotoxin-resistant sodium channel selectively expressed in nociceptive dorsal root ganglion neurons, where it regulates action potential threshold and repetitive firing (96). PI3 K inhibition suppresses TRPV1 upregulation in bone cancer pain (97), and PI3K-Akt-mTOR signaling promotes structural and synaptic plasticity (98), including Akt-dependent upregulation of Nav1.8 as shown in a non-cancer inflammatory model (96) and TNF-α-driven Nav1.8 upregulation (99), thereby lowering action potential thresholds.

### 5.4 TNF receptors at the tumor-nerve interface

Perineural invasion is common in pancreatic, head and neck, and prostate cancers and is associated with greater pain severity (23, 24). Tumor-nerve contact is active rather than passive: neurotrophic and axon-guidance programs remodel intratumoral innervation, and macrophage-nerve interactions drive neuroinflammation in a prostate cancer model of perineural invasion (23). Within this interface, TNFR1 expression on tumor cells mediates immune escape and an immunosuppressive microenvironment (100), and TNFR1 signaling promotes tumor growth in pancreatic and ovarian cancer models (101, 102). Metastatic oral cancers from patients with pain overexpress candidate pain-associated oncogenes whose products are released in exosomes (103). Except for the perineural-invasion model cited above, these data come from tumor models without pain endpoints; whether TNF-dependent axon-guidance signaling increases nerve density or sensitizes primary afferents remains untested.

Thus, TNF-α engages NF-*κ*B, MAPK, and PI3 K/Akt through TNFR1/TNFR2. These pathways have distinct functional emphases—transcriptional amplification, glial activation, and direct neuronal sensitization—but operate in crosstalk to form a coordinated signaling network. However, combined deletion of TNFR1 and TNFR2 in a fibrosarcoma model suppressed tactile hypersensitivity while accelerating tumor growth (56), suggesting dual pro-nociceptive and tumor-suppressive functions of TNF-α signaling. Current mechanistic evidence derives primarily from bone cancer and chemotherapy-induced pain models; the relative contribution of these pathways has not been compared across cancer pain subtypes. Notably, the mechanistic evidence reviewed in Sections 5-7 derives largely from single-sex (predominantly male) designs, so sex-dependent cytokine signaling in cancer pain remains untested.

## 6 Signaling mechanisms of IL-6 in cancer pain

IL-6 is a four-helical bundle cytokine produced by tumor cells, infiltrating immune cells, and stromal cells (55); within the CNS, glial and endothelial cells are the principal sources (104). In contrast to the adaptor-recruited TNFR system, IL-6 signals through a JAK-associated receptor complex comprising the ligand-specific IL-6 receptor (IL-6R) and the shared signal transducer gp130. Membrane-bound IL-6R (mIL-6R) has restricted expression and mediates classical signaling (105, 106), whereas soluble IL-6R (sIL-6R), generated by proteolytic shedding or alternative splicing, is widely distributed in body fluids and the tumor microenvironment and mediates trans-signaling (107). Both modes require gp130 engagement to activate downstream cascades (108). Because gp130 is shared by other IL-6 family cytokines, including CNTF and IL-11 (109), IL-6 signaling in cancer pain may be inherently convergent or synergistic with related ligands. Ligand-receptor complex formation activates receptor-associated JAK kinases, predominantly JAK1 and JAK2, which then engage three major downstream pathways: STAT3, MAPK, and PI3 K/Akt (110). These pathways share a common JAK node but differ in functional output and exhibit crosstalk, forming a branched signaling network.

### 6.1 STAT3: transcriptional regulation

Activated JAK phosphorylates tyrosine residues in the gp130 intracellular domain, recruiting STAT3 for phosphorylation and nuclear translocation, where STAT3 induces pro-nociceptive genes including IL-1β and NGF, directly amplifying nociceptive signaling (111) (Figure 2). In a rat model of oxaliplatin-induced acute neuropathic pain, spinal IL-6 and p-STAT3 are concurrently elevated (112); in a breast cancer bone pain model, STAT3 inhibition significantly alleviates pain behaviors and downregulates IL-6 and IL-1β mRNA (113), supporting a pathogenic role for STAT3 in cancer pain. A bidirectional positive feedback loop exists between IL-6 and STAT3, as STAT3 can also promote IL-6 transcription; however, the contribution of this amplification loop to cancer pain chronification remains to be tested across different time windows.

**Figure 2:**
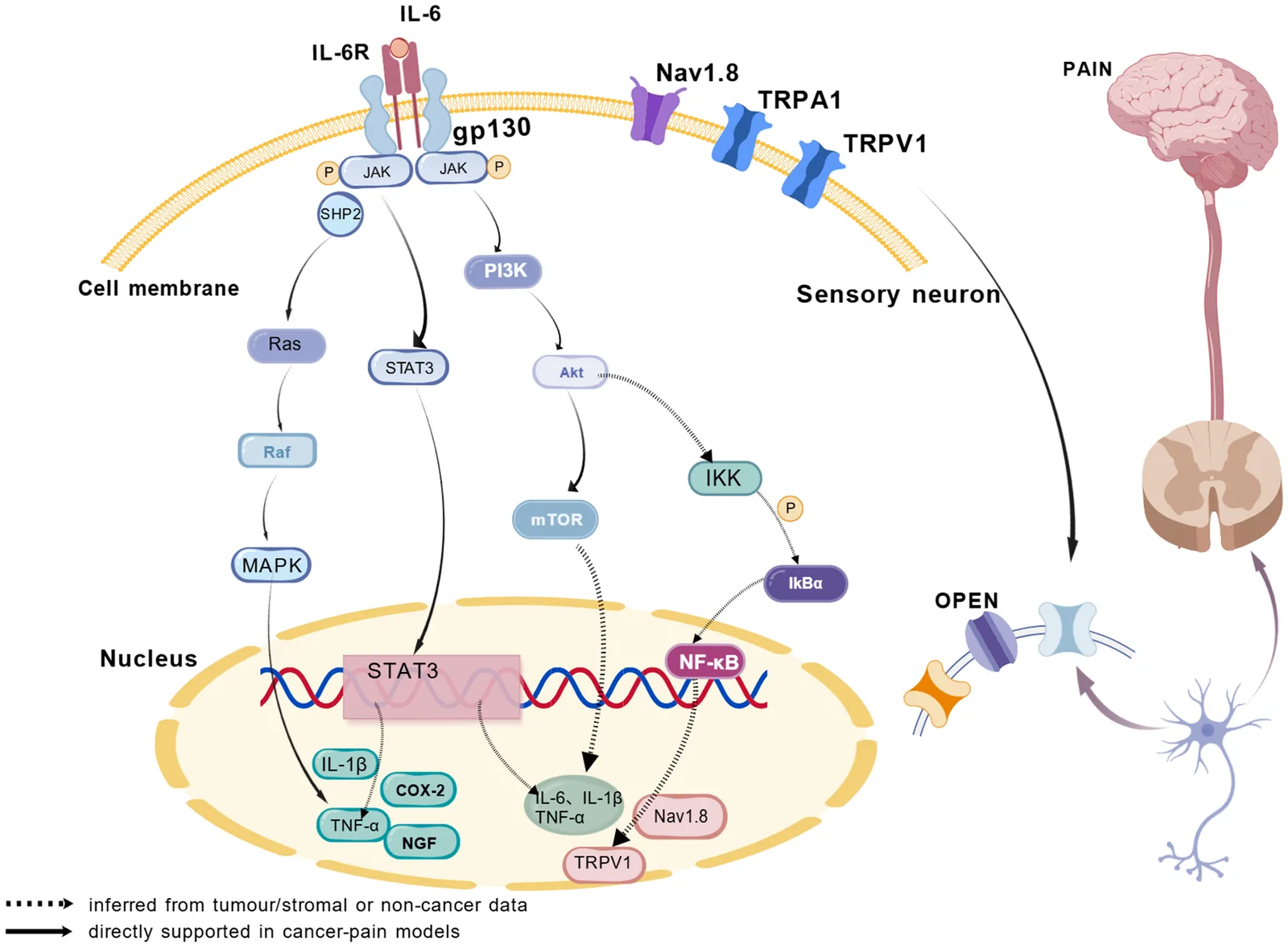
IL-6 signaling in cancer pain. Figure 2 schematically depicts classical IL-6 signaling through membrane-bound IL-6R on dorsal root ganglion and spinal dorsal horn neurons; trans-signaling via soluble IL-6R is supported in bone cancer pain but is not shown. This is a composite schematic drawing on neuronal, glial, tumor, and stromal evidence; not every arrow is established specifically in sensory neurons. IL-6 binding to IL-6R/gp130 activates JAK, which drives STAT3, Ras–Raf–MAPK, and PI3K–Akt–mTOR. Akt phosphorylates IKK to promote I*κ*B*α* degradation and NF-*κ*B activation. These cascades upregulate pronociceptive mediators and ion channels (Nav1.8, TRPV1, TRPA1). STAT3-linked regulation of TNF-α and other mediators is depicted as inferred where direct cancer-pain evidence is lacking. OPEN marks the open (activated) channel state. Solid arrows denote signaling nodes or functional relationships for which direct evidence exists in cancer-pain models. Dashed arrows denote upstream receptor–pathway connections, inferred relationships, or evidence derived from non-cancer, tumour, or stromal studies. A solid arrow downstream does not imply that the entire upstream receptor-to-effector sequence has been demonstrated in cancer pain. Created with BioGDP.com (150). IL-6R, IL-6 receptor; gp130, glycoprotein 130; JAK, Janus kinase; STAT3, signal transducer and activator of transcription 3; MAPK, mitogen-activated protein kinase; PI3 K, phosphoinositide 3-kinase; Akt, protein kinase B; mTOR, mechanistic target of rapamycin; IKK, I*κ*B kinase; I*κ*B*α*, inhibitor of NF-*κ*B *α*; NF-*κ*B, nuclear factor *κ*B; TRPA1, transient receptor potential ankyrin 1; TRPV1, transient receptor potential vanilloid 1; Nav1.8, voltage-gated sodium channel 1.8.

### 6.2 MAPK and PI3 K/akt: neuronal sensitization

#### JAK/MAPK branch

In addition to STAT3, JAK-mediated phosphorylation of gp130 Tyr759 recruits SHP2, which activates the Grb2-SOS-Ras cascade and downstream MAPKs. In a rat bone cancer pain model, inhibition of TNF-α synthesis with pentoxifylline and gp130-associated signaling with SC144 reduced p38 and JNK phosphorylation in the dorsal root ganglia (59), implicating the JAK/MAPK branch in the neuropathic component of bone cancer pain.

#### JAK/PI3 K/Akt branch and convergence with NF-*Κ*b

JAK activation also phosphorylates the PI3 K regulatory subunit p85, generating PIP3 and recruiting Akt to the membrane for activation (114). In the periaqueductal gray (PAG) of a breast cancer bone pain model, p-PI3 K, p-Akt, and p-mTOR were upregulated together with IL-6 and its receptors, and the gp130 inhibitor SC144 attenuated mechanical and thermal hyperalgesia while downregulating p-PI3 K and p-mTOR (58), indicating that gp130-dependent signaling enhances neuronal excitability through PI3 K/Akt/mTOR signaling. Activated Akt further phosphorylates IKK, inducing I*κ*B*α* degradation and NF-*κ*B nuclear translocation, thereby establishing an IL-6/PI3 K/Akt/NF-*κ*B pathway (115, 116). In Walker 256 tibial cancer pain, morphine has been proposed to modulate DRG neuronal apoptosis and the inflammatory microenvironment through this route (117). This branch provides downstream convergence with TNF-α signaling at NF-*κ*B, explaining how distinct upstream cytokines can generate overlapping inflammatory and pro-nociceptive transcriptional programs.

Collectively, IL-6 engages STAT3, MAPK and PI3 K/Akt-NF-*κ*B through gp130-dependent JAK activation. Although most interventions in cancer-pain models do not distinguish classical from trans-signaling, Fang et al. (129) directly demonstrated IL-6/sIL-6R trans-signaling in a rat bone cancer pain model; its relative importance and generalizability remain unresolved. Current evidence derives mainly from bone cancer pain and chemotherapy-induced pain models, and the relative contribution of these branches across cancer pain subtypes has not been compared; the immunological safety of gp130-directed intervention has not been examined in this setting.

## 7 Cross-regulation of TNF-α and IL-6 in cancer pain

TNF-α and IL-6 are frequently co-elevated in the tumor microenvironment and nervous system, and each cytokine independently promotes pain; however, whether their combined effect exceeds the sum of their individual effects has not been tested in cancer pain. In rat cancer-induced bone pain, spinal TNF-α mRNA rises on day 6, preceding IL-6 upregulation on day 12 (119). This sequence is compatible with TNF-α initiating and IL-6 sustaining nociceptive signaling, but timing alone does not establish such a hierarchy, which would require phase-specific interventions. The only comparison of combined versus single-agent blockade comes from collagen-induced arthritis, in which a dual TNF-α/IL-6 fusion protein inhibited osteoclastogenesis and bone erosion more effectively than monotherapy (120); that study assessed inflammatory, cellular, and structural outcomes rather than pain. Mechanistically, the two signaling networks intersect at multiple levels—reciprocal transcriptional amplification, shared signaling nodes, and convergent pathological endpoints—thereby forming a self-amplifying inflammator*y* axis (Figure 3), a term we use to denote these intersections rather than a demonstrated feedback circuit.

**Figure 3:**
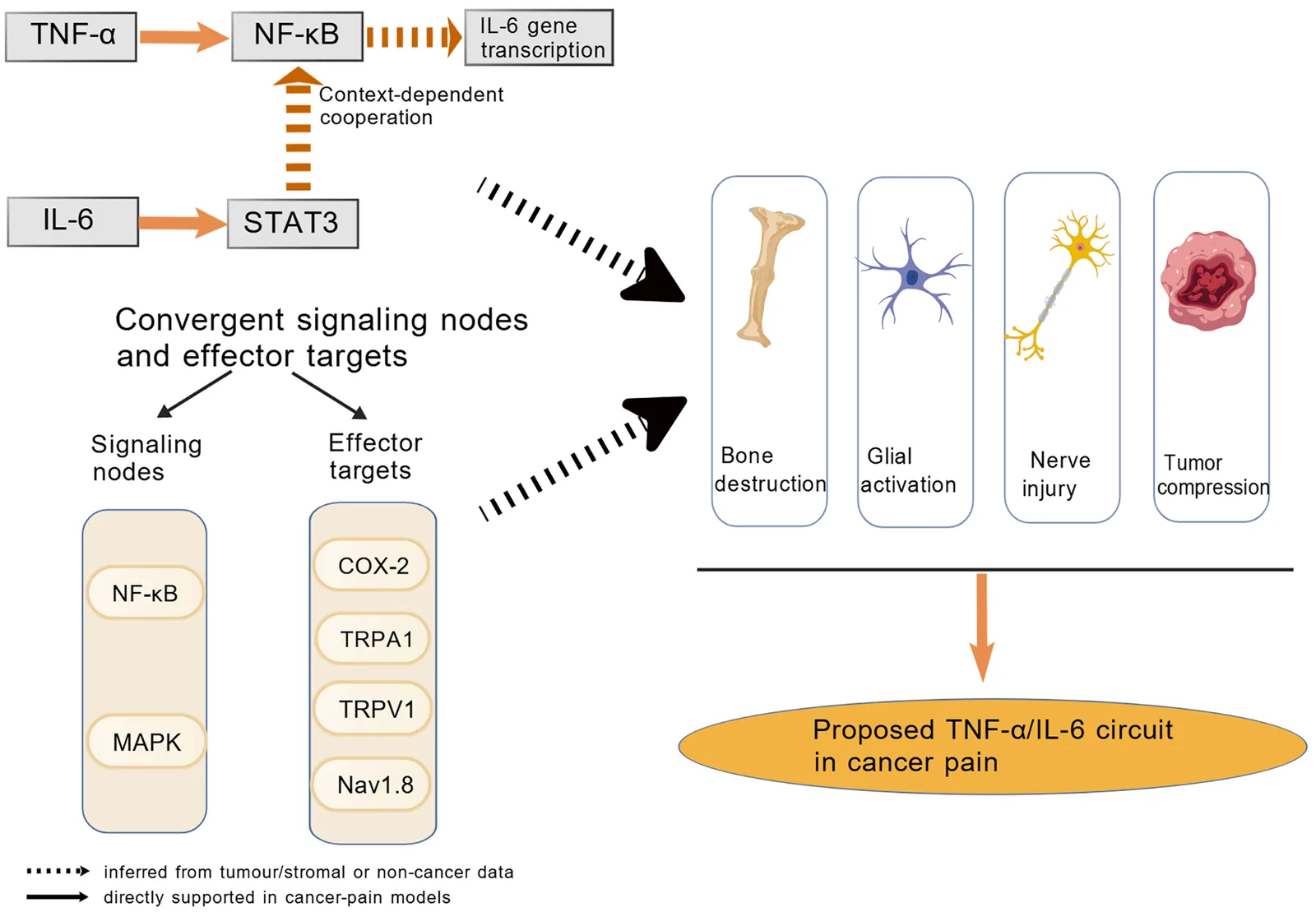
Proposed TNF-α/IL-6 circuit in cancer pain. We propose that TNF-α–NF-*κ*B and IL-6–STAT3 may form a positive-feedback loop: NF-*κ*B drives IL-6 transcription, and STAT3 may enhance NF-*κ*B activity through context-dependent mechanisms. The circuit converges on shared signaling nodes (NF-*κ*B, MAPK) and effector targets (COX-2, TRPA1, TRPV1, Nav1.8). Dashed arrows link the proposed circuit to bone destruction, glial activation, nerve injury, and tumor compression. This figure presents a proposed inflammatory interaction network inferred mainly from tumour and stromal data; it does not depict demonstrated synergistic analgesia. Solid arrows denote signaling nodes or functional relationships for which direct evidence exists in cancer-pain models. Dashed arrows denote upstream receptor–pathway connections, inferred relationships, or evidence derived from non-cancer, tumour, or stromal studies. A solid arrow downstream does not imply that the entire upstream receptor-to-effector sequence has been demonstrated in cancer pain. Arrow color distinguishes ligand-specific pathways (orange, TNF-α/IL-6; black, downstream signaling) and does not indicate evidence strength. Created with BioGDP.com (150). NF-*κ*B, nuclear factor *κ*B; STAT3, signal transducer and activator of transcription 3; MAPK, mitogen-activated protein kinase; I*κ*B*α*, inhibitor of NF-*κ*B *α*; COX-2, cyclooxygenase-2; TRPA1, transient receptor potential ankyrin 1; TRPV1, transient receptor potential vanilloid 1; Nav1.8, voltage-gated sodium channel 1.8.

### 7.1 Reciprocal transcriptional amplification

TNF-α and IL-6 can induce each other transcriptionally, forming a reciprocal, potentially self-amplifying transcriptional loop that, if operative in nociceptive tissues, would represent the most upstream level of their interaction. TNF-α-TNFR1 signaling activates NF-*κ*B, and nuclear p65/p50 directly initiates IL-6 transcription (121, 122). In myeloma stroma, TNF-α promotes IL-6 release from fibroblasts (123); in melanoma, TNF-α-TNFR1 signaling directly drives IL-6 transcription and is linked to mitochondrial dysfunction and senescence (124). These findings position TNF-α as an upstream inducer of IL-6 in the tumor microenvironment rather than merely a co-expressed cytokine. Conversely, IL-6-activated STAT3 may cooperate with NF-*κ*B to sustain TNF-α expression, an interaction described as an “IL-6 inflammatory amplifier” (125); however, the precise molecular mechanism in cancer-pain tissues has not been established. Promoter-based and tumour/stromal studies suggest that NF-*κ*B and STAT3 can reciprocally regulate IL-6 and TNF-α in non-neuronal contexts (121, 123–125), but direct evidence for this circuit in sensory neurons or spinal glia is lacking. The proposed reciprocal TNF-α/IL-6 transcriptional loop in cancer pain therefore remains inferred, and whether its sustained operation contributes to persistent nociceptive amplification has not been tested. Its real-time dynamics in dorsal root ganglia or spinal cord and its phase-specific contribution remain to be established through time-series sampling and targeted blockade.

### 7.2 Shared signaling nodes and convergent effectors

Beyond transcriptional crosstalk, TNF-α and IL-6 converge on shared signaling nodes—principally NF-*κ*B and MAPK—and on common pain-related effectors. COX-2 exemplifies this convergence. TNF-α-NF-*κ*B signaling promotes COX-2 transcription through NF-*κ*B binding to the COX-2 promoter (126), whereas IL-6 activates COX-2 via JAK/STAT3; in lung cancer cells, IL-6-induced COX-2 upregulation is blocked by anti-IL-6 antibody and is STAT3-dependent (127). The COX-2 promoter contains both NF-*κ*B and STAT response elements (128), providing a structural basis for cooperative transcription, although whether combined TNF-α/IL-6 stimulation produces nonlinear transcriptional enhancement in DRG neurons or spinal glial cells has not been tested.

Ion channels represent a second convergent effector class. In the dorsal root ganglia of rats with bone cancer pain, TNF-α, IL-6, TNFR1, IL-6R, and TRPA1 are concurrently upregulated; pentoxifylline and SC144 attenuated mechanical and thermal hyperalgesia and reduced TRPA1 protein and p38/JNK phosphorylation in the dorsal root ganglia (59). These data support convergence of TNF-related and gp130-associated signaling on TRPA1 via p38/JNK; however, receptor-specific causality and the extent to which this convergence sustains peripheral sensitization require selective dual-blockade studies. TRPV1 and voltage-gated sodium channels Nav1.7/Nav1.8 are similarly co-regulated: TNF-α increases TRPV1 transcription via NF-*κ*B (81), whereas IL-6 trans-signaling, specifically the IL-6/sIL-6R complex, enhances DRG excitability through JAK/PI3K-dependent TRPV1 upregulation (129), and IL-6 also increases Nav1.7/Nav1.8 activity in DRG neurons (130). Because gp130 and JAK are shared by multiple IL-6-family cytokines, this convergence reflects a shared downstream mechanism rather than an IL-6-exclusive pathway. Thus, both cytokines lower nociceptor thresholds by targeting the same effector channels, and whether their combined regulation is additive or synergistic requires dual blockade in the same experimental system.

### 7.3 Convergent pathological endpoints and proposed cooperative effects

At the pathological level, TNF-α and IL-6 converge on shared processes—bone destruction, glial activation, nerve injury, and tumor compression—but whether their combined effects exceed those of individual cytokines in cancer pain remains untested; most data derive from single-cytokine studies, and dual-intervention experiments are scarce.

Bone destruction is a major driver of advanced cancer pain. TNF-α induces RANKL and suppresses osteoprotegerin via NF-*κ*B (131, 132), whereas IL-6 promotes RANKL expression via STAT3 (118). In collagen-induced arthritis, dual TNF-α/IL-6 targeting with DTA improved anti-inflammatory and structural outcomes compared with monotherapy (120). Because no formal interaction analysis was performed, this does not establish synergy or an effect exceeding additivity; whether this joint effect extends to tumor-induced osteolysis and associated pain remains untested.

In the CNS, TNF-α polarizes microglia toward a pro-inflammatory state and stimulates IL-1β and IL-6 release. IL-6, in turn, sustains microglial activation and promotes astrogliosis, maintaining algogenic mediator release and central sensitization (133–135). Because microglial function reflects a dynamic balance between pro- and anti-inflammatory states rather than a binary switch (134), sustained TNF-α/IL-6 signaling may shift this balance toward the pro-inflammatory phenotype (136). These observations suggest a glial-neuronal amplification loop, but direct dual-blockade experiments are required.

In vincristine-induced neuropathy, TNF-α elevation in the spinal cord accompanies glial activation and pain behavior (137). The link to myelin comes mainly from peripheral nerve injury models, where TNF-α upregulates MMP-9 via NF-*κ*B to degrade myelin and recruit macrophages in a non-cancer model (138); whether the same sequence operates in chemotherapy-induced neuropathy has not been shown. IL-6 signaling may cooperate with NF-*κ*B in a context-dependent manner to sustain TNF-α expression (125), but the precise mechanism in cancer-pain tissues has not been established. In tumor compression-related pain, TNF-α-driven tumor proliferation and IL-6-mediated edema may converge on mechanosensitive channels such as PIEZO (139), but this remains pathway inference without functional validation.

In a tumor-biology study of non-small cell lung cancer without pain endpoints, IL-6 and TNF-α promoted epithelial-mesenchymal transition, and higher expression of both cytokines was associated with lymph-node and distant metastasis (140). Their elevation may therefore reflect both more advanced disease and stronger nociceptive drive, which existing datasets cannot separate. Whether TNF-α and IL-6 promote cancer pain independently of tumor stage remains undetermined because tumor burden is seldom reported or controlled.

Collectively, TNF-α and IL-6 may have the potential for greater-than-additive interaction across multiple pathological processes, but this remains a testable hypothesis in cancer pain. Direct evidence is strongest for transcriptional positive feedback and dual-target efficacy in arthritis models; in cancer pain, synergy remains largely inferred from single-cytokine studies. We therefore propose that TNF-α/IL-6 synergy in cancer pain is a testable hypothesis. Future studies should incorporate single-agent and combination arms at defined doses, with a prespecified interaction analysis against an explicit additive expectation, into bone cancer pain and chemotherapy-induced pain models, combining pain behavior with signaling phosphorylation dynamics and transcriptional profiling to determine whether combined inhibition produces effects greater than the sum of monotherapies. Behavioral readouts should span both evoked responses (von Frey, Hargreaves) and spontaneous pain (place preference, guarding), and studies must report and exclude sedation or motor impairment as scoring confounders. Additionally, because analgesia in cytokine-blockade studies may be secondary to reduced tumor burden or attenuated osteolysis rather than direct neuronal effects, tumor-volume and osteolysis controls are needed to disambiguate mechanism in both single-agent and combination experiments.

## 8 Therapeutic implications and translation

Preclinical evidence supports the feasibility of targeting the TNF-α/IL-6 axis in cancer pain. Etanercept reduced thermal and mechanical hyperalgesia in a bone metastasis-induced cancer pain model (141), and IL-6 blockade attenuated pain behavior and pathological bone remodeling in a rat model of bone metastasis-induced cancer pain (118). In humans, the evidence for TNF-α blockade in cancer pain is limited to two uncontrolled case reports of perispinal etanercept in patients with spinal metastases from lung or breast cancer (142). These reports described pain relief, weight gain, and reduced PET metabolic activity, but without controls, they provide only limited observations on the administration and reported tolerability of perispinal etanercept; they do not establish general safety or tolerability, analgesic efficacy, or antitumor activity. Tocilizumab is approved for cytokine release syndrome and other non-cancer indications (143), which shows that IL-6 blockade can be delivered in oncology but provides no evidence of efficacy in cancer pain. Importantly, therapeutic interpretation must account for receptor specificity. Etanercept and infliximab neutralize both soluble and transmembrane TNF, engaging TNFR1 and TNFR2, whereas soluble-TNF-selective agents such as XPro1595 spare transmembrane TNF-TNFR2 signaling (91). Because TNFR2 can exert context-dependent protective effects and TNF signaling may restrain tumor growth (56), the choice between non-selective and soluble-TNF-selective strategies cannot be based on analgesic considerations alone.

Indirect evidence comes from conventional analgesics that modulate these cytokines, but each has limitations. NSAIDs attenuate prostaglandin-dependent inflammatory signaling, but long-term use is limited by cardiovascular and gastrointestinal toxicity (144). In preclinical models, morphine produces analgesia yet can promote TNF-α-dependent neuroinflammation through Toll-like receptor 4 (145) and induce intestinal inflammation and gut dysbiosis (146). Dexmedetomidine suppresses TNF-α and IL-6 via MAPK/NF-*κ*B but is limited by hypotension and bradycardia (147). These trade-offs highlight the appeal of more selective, mechanism-based blockade, but they do not establish cytokine blockade as a clinically validated alternative to opioids.

However, single-target efficacy likely depends on the degree to which pain remains inflammation-dependent; once neuropathic remodeling or self-sustaining central sensitization predominates, blocking TNF-α or IL-6 alone may be insufficient. Dual targeting is mechanistically attractive given the transcriptional positive feedback loop and downstream convergence, but safety concerns remain: TNF-α inhibition increases tuberculosis reactivation risk (148), IL-6 blockade may mask infection (149), and cancer pain patients are frequently immunocompromised. Local or time-limited delivery has been proposed as a risk-reduction strategy, but its efficacy and safety remain unproven in this setting.

Current evidence therefore defines a logical starting point, not a validated endpoint, for non-opioid strategies targeting this axis. Cancer pain-specific trials, particularly those using local administration or time-limited dual blockade, are needed to determine whether an acceptable balance between analgesic benefit and systemic immunosuppression can be achieved. The evidence reviewed does not support routine use of TNF-α/IL-6 blockade solely for cancer-pain treatment; its role should be confined to appropriately designed clinical studies.

## 9 Conclusions and future perspectives

We propose a conceptual shift from viewing TNF-α and IL-6 as parallel pro-nociceptive mediators to an integrated, putatively self-amplifying axis in cancer pain. The integrated circuit and its proposed effects on cancer pain are inferred relationships that require experimental validation. Proposed transcriptional positive feedback, convergence on NF-*κ*B/MAPK/PI3K-Akt, and shared regulation of COX-2, TRPV1, TRPA1, and Nav1.8 may couple this axis to bone destruction, glial activation, nerve injury, and tumor compression. Although preclinical blockade of either cytokine attenuates pain-related behavior, translation is limited by two unresolved issues: whether single-target inhibition remains effective once neuropathic remodeling and central sensitization predominate, and whether dual blockade can achieve analgesia without unacceptable immunosuppression. Current evidence relies on single-sex, largely preclinical designs; sex-stratified validation is therefore required before clinical translation. Comparative single-agent and combined-blockade designs with prespecified interaction analysis remain the critical next step.
